# Joining with the Conversation: Research as a Sustainable Practice in the Sport Sciences

**DOI:** 10.1186/s40798-022-00493-0

**Published:** 2022-08-06

**Authors:** Carl T. Woods, Duarte Araújo, Keith Davids

**Affiliations:** 1 Institute for Health and Sport, Victoria University, Melbourne, Australia; 2 CIPER, Faculdade de Motricidade Humana, Universidade de Lisboa, Cruz Quebrada, Lisbon, Portugal; 3 Sport and Human Performance Research Group, Sheffield Hallam University, Sheffield, UK

**Keywords:** Correspondence, Research processes, Ecological, Ontology, Knowing, Skill, Sustainability

## Abstract

What would it mean to consider research in the sport sciences as a sustainable practice? Taking a step back, in such a context, what would sustainability even mean? The time is ripe to address such questions, and what we lay out here are our initial thoughts on this most contemporary of issues. We start by exploring what is meant by the term ‘sustainability’. Rather than following mainstream thinking—the harnessing of earthly resources commodified and exploited as ‘renewables’—we situate it in the sport sciences as a continuing *response-ability to the experiences of others.* This view is rooted in ‘commoning’—an intransitive verb in which people conjoin varied experiences through correspondence. What makes this sustainable, is its ongoing open-endedness; meaning, it *carries on* as people (co)*respond* to one another. Central to this idea is a perceptual system attuned to the ebbs and flows of what or who one is corresponding with. Though, the current modus operandi of research in the sport sciences is located, not on the skilled perception of the scientist corresponding with the coming-into-being of phenomena, but on an unsustainable model of recognition that views phenomena as ‘objects of analysis’, fixed and final in form, waiting to be known *about* by means of reduction, fragmentation and classification. For research in the sport sciences to become a sustainable practice, we propose a scholarship that prioritises direct observation and participation with what holds our attention, corresponding within its natural ecology of relations, embedding the phenomenon itself. This re-conceptualisation of science views research as a *response-able scholarship grounded in conversation*. Like inquiring about the well-being of loved ones, what sustains such a scholarship is *curiosity, care* and *hope*—a curiosity about which captivates us, a care that sees us respond to what we observe, and a hope of carrying the correspondence on, together.

## Key Points

- We propose a view of sustainability in the sport sciences as a *response-ability to the experiences of others*. This position is rooted in ‘commoning’—an intransitive verb in which people conjoin experiences through correspondence. What makes this sustainable, is its ongoing open-endedness; meaning, it *carries* *on* as people (co)*respond* to one another.
- Central to this idea is a perceptual system attuned to the ebbs and flows of what or who one is corresponding with. Meaning, it is the *direct perception* of the scientist that leads them to grow knowledge *of* the phenomena they study *with*.
- For research in the sport sciences to become a sustainable practice, we propose a scholarship that prioritises direct observation and participation with what holds our attention, corresponding within its natural ecology of relations. That is, a *response-able scholarship grounded in conversation*.

A satisfying conversation is neither rigidly programmed nor chaotic; somewhere between perfect order and total surprise we look for a creative tension; a progressive and mutual deepening of insight, a sense that we are getting somewhere worthwhile—Stephen L. Talbott

## Prologue: A Plea

In writing *The Crisis in Education*, philosopher Hannah Arendt [1] proclaimed that at some point, people must decide whether they care enough about the world to assume responsibility for it. As sport scientists, we care about where our discipline is placed, the directions it could be heading, and what may lay ahead for those to come. This paper, then, is our attempt to follow Arendt, and assume some responsibility for these things by proposing what research in the sport sciences could entail if it were to be undergone as a *sustainable practice*.

Indeed, in the supposed epoch of the Anthropocene, it is hard to find a theme as universally important as that of sustainability. National governing bodies, educational institutions, and many local organisations seem fixated on finding ways to turn earthly resources—wind, sunlight, water—into commodities to preserve a Western lifestyle fundamentally rooted in continued high levels of materialist consumption. We must confess, this is not a view of sustainability that has ever sat well with us. It risks a human-centric notion of control, manipulation, and hierarchy—a deep seated desire to modify and exploit the surrounding environment, viewed only as a resource, to further a way of life replete with social and ecological injustice; one that has led entire ecosystems to the brink of collapse, and pushed others well beyond. We are reminded of the cautionary words of the ecological psychologist James Gibson [2], in his seminal text *The Ecological Approach to Visual Perception* (p. 130):

“There is only *one world*, however diverse, and all animals live in it, although we human animals have altered it to suit ourselves. We have done so wastefully, thoughtlessly, and if we do not mend our ways, fatally.”

The view of sustainability we cast forward is one which sees us learn to become *response-able to the experiences of others*. This perspective raises important questions for sport scientists—namely, how do we contribute to the continuity of sport science and ensure we harbour conditions that support future researchers and practitioners in carrying on with their journey? As sport scientists, *response-ability* would involve genuinely *listening* to what others (i.e. coaches, athletes, scientists and practitioners) have to share with us. This is not just to extract ‘hard facts’ represented as ‘data’ to be retrofitted into traditional disciplinary frameworks, supporting prior established hypotheses, but to join *with* others in their story of becoming, moving along with their direction of travel, together. Such an approach moulds answers into further questions—questions that do not *close in* on a definitive, all-encompassing ending, but that *open up* to continued exploration, leading to further opportunities *for all* to carry on [3]. Central to this view of sustainability is a relational, pragmatic philosophy [4]—a dynamic way of seeing, hearing and feeling in which one observes and participates with what holds their attention, responding with care and curiosity. Like entering into a conversation with someone we are especially fond of, joining a flow of research undergone sustainably is not vertical; an imposition of hypothesis down onto motionless matter, but *longitudinal*; a going along with guided by attentive and active responsivity.

As sport scientists, sustainability may not be a topic that colleagues consider to be high on a research agenda. In fact, ‘development’ and ‘performance’—concepts sport science traditionally focuses on in research and practice—have even been argued as being counterintuitive to certain views of sustainability [5]. But should this mean we shy away from addressing such a topic? Sport, after all—inclusive of its athletes, practitioners, philosophers, scientists, technologists, broadcasters, spectators, and numerous others—is a relational phenomenon that is *of* this world; a world that we owe our very existence to; a world that calls for our attention. Thinking that the topic of sustainability resides in ‘a world outside of our professional discipline’ would, to us at least, be irresponsibly naive at best, and irreversibly damaging at worst. Thus, this piece should be read as *a plea*; a plea for sport science to venture beyond its artificial bounds and look towards what those outside of our conventional ‘scope’ can share with us, and we with them. Of course, some may think that such a plea is overly esoteric or ‘too philosophical’ for the ‘hard science’ that is often synonymous with sport performance. We would simply remind such readers that we are all responsible for a world that is older than us; a world that will outlive us reading these very words; a world that is not exceptionally ‘ours’, but one, as James Gibson noted, that we *share*.

## Introduction

What would it mean to think of research as a sustainable practice in the sport sciences? Moreover, in the context of sport science research, what would sustainability even mean? The time is ripe to ask such challenging yet important questions, and what this paper presents, is our attempt to answer them. However, we humbly profess that the position provided is far from complete. It may even open up more questions, thereby inviting others to join in with our conversation, carrying it on while moving along their direction of travel.

We start by discussing what is meant by the well-used word, ‘sustainability’. Without context, this is a word that risks abstraction [6], perhaps added to the policies and documents of national governing bodies to ‘greenwash’ a decision by giving an outward facing appearance of being ‘contemporarily-’, ‘environmentally-’, ‘socially-’, ‘economically-’, or ‘ethically-’ minded ([3], ch. 21). Moreover, in the current techno-scientific proliferation, it is a word many may view synonymously with the implementation of technology designed to mine, harvest, control, and preserve planetary resources, coined as ‘renewables’. We want to suggest, though, that these views of sustainability not only risk ambiguity, abstraction, and techno-fixity, but exceptionalism and discontinuity, encouraging us to strive towards a humanly focused totality that leads to closure, not openness. Drawing on anthropologist Tim Ingold’s [7] notion of ‘commoning’, we argue that sustainability is less about reaching a mythical, circular steady-state, and more about growing a *response-ability to the experiences of others so that, together, we can find reliable and longstanding ways of carrying on*.

Framed as such, how are we to regard knowledge stemming from research as a sustainable practice in the sport sciences? Answering this question requires a profound epistemic shift—that being: knowing is *ecological*, not (re)cognitive. By this, we mean that to *really* know phenomena we study, we need to observe and primarily experience things *relationally*, in their natural ecology of relations, not as final or fixed forms detached or separated from them [4]. Such relationality requires a deep embeddedness *within* the context in which phenomena emerge, leading researchers to observe and participate with constituents as an inhabitant, not document or interpret them as an occupant, mediated through some representational or conceptual lens [8–11]. The growth of knowledge viewed ecologically, then, does not occur through a model of categorical *recognition*—establishing a homological match between structures of the mind and structures of the world—but through *direct perception*, a progressive attunement to the sights, sounds, smells, tastes and feels of what captivates us [2, 4, 10]. Typically, it is the former that dominates research paradigms within the applied sciences, including neurocomputational psychology (e.g. [12]) and sport science (e.g. [13]). This domination is manifest in the classification and labelling of phenomena viewed ‘objectively’, perpetuated through the hypothetico-deductive theory of the scientific method [14]. It has led to an imbalance that detaches the researcher from the research [8], where phenomena need to be viewed ‘objectively’, oft-represented as data to be mined, modelled and classified away into disciplinary frameworks [11, 15]. To over-rely on this perspective is to turn our back on the *becomings* of things, where the main goal is to know *more*—i.e. ‘filling gaps within our scientific knowledge base’—not *better*—i.e. developing a progressive sensitivity to the becoming of phenomena-in-place. Leaning on key ideas from ecological psychology [2, 16], we argue that this dichotomy of *how to know* is an issue of traditional scholarship prioritising knowledge *about*, not knowledge *of*, the environment.

How, then, are we to take up with research practised *response-ably* in the sport sciences, thereby prioritising the direct perception of the researcher in coming to know (*of*) what they study? To answer this question, we weave in the ideas of Johann Wolfgang von Goethe (1749–1832); work which encourages a view of scientific inquiry *as a conversation* [17–19]. This perspective offers us a different way of approaching phenomena, a way that could help overcome the more dominant hard empiricism pervasive to the sport sciences; replacing surprise with astonishment, prediction with anticipation, closings with openings. Like entering into a conversation with someone we are especially fond of, what sustains research-as-a-conversation in sport science is *curiosity*, *care* and *hope* [7]. It is a curiosity which sees us continually attend to the well-being of what sparks our interest, a care that sees us skilfully respond to what we find, and a hope that together, we can carry the correspondence on. Thus, for research to be practised sustainably in the sport sciences, we propose a *response-able scholarship ground in ongoing conversation*.

## Sustainability *as* Response-Ability

Indeed, the notion of sustainability can be somewhat paradoxical ([3], ch. 21). On the one hand, it evokes a sense of limit—an ending, a closure, a totality. This is a view that sees the resources of the world as commodities, continually dwindling in their capacity to sustain a humanly exceptional way of life, fundamentally challenged by continued over-consumption. Yet on the other hand, it evokes a sense of limitlessness—a circularity, a perpetual renewal—a view that strives towards attaining a steady-state that neither extends our reach, nor over-steps the bounds of consumption ([3], ch. 21). How, then, are we to give meaning to a notion that seems to be about acknowledging both an end and a renewal? Unfortunately, over-viewing countless documents, policies and strategic development plans of many national governing bodies, educational institutions and local organisations offers little guidance here. Despite being increasingly plastered across such things, the word ‘sustainability’ is often presented in a contextless and ambiguous way.^1^ This leaves us with the rather pessimistic view that it is a word simply used to give a ‘contemporarily-minded’ outward facing appeal, yet inwardly, the status quo remains unchanged, progressing on with business as usual.

Some may argue that it is unfair for us to be overly critical here, as despite its frequent appearances, there has been little thought that provides grounding as to what sustainability *actually means*, both in policy and practice [6]. To this point, it has even been suggested that by the late 1990s, there were definitions of sustainability reaching into the thousands [20]—ranging from ‘environmental protection’ to ‘sustainable economic growth’ [21]. Where does this leave us, then, in our quest to situate research as a sustainable practice in the sport sciences?

To address this question, we introduce the word ‘commoning’; an intransitive verb which anthropologist Tim Ingold [7] discusses in his book, *Anthropology and/as Education*. Borrowing this phrase from Menzies [22], Ingold situates it as a means of actively joining with the varied experiences of others encountered in life, leading us to find ways of carrying on, together. This is because “having in common”, according to Ingold ([7], p. 6), is not a prerequisite of life, but an aspiration people must continue to work at, responding and opening up to one another; old and young, mature and immature. As such, there is no end-point in commoning, nor is there a one-sided, pre-defined agenda to be transmitted to some passive recipient. Rather, in commoning, “ends are as yet undefined and undefinable, beyond the horizons of conceptualisation, and for that very reason, they remain open to all” ([7], p. 38).

For us, the crucial part of commoning is the requisite *responsivity*—it can only occur if people are open and (co)respond to the experiences of one another [7]. Take this very paper, for example, throughout its inception, I (the first author) responded to the suggestions offered by co-authors about its direction of travel, as they responded to what I laid out in various drafts. This active responsiveness led to the growth of what you are currently reading; growth which we did not fully perceive from the outset, but that emerged as we carefully attended and responded to the comments and suggestions put forth by all active participants (i.e. authors, journal editors, and reviewers). In other words, what you are reading is not the output of a fully formed idea closed off from the world, predetermined by one member of the authorship that was waiting to be passively ‘typed up’. It is an emergent mesh of the ideas, perspectives and experiences cast forward by all, conjoined through our ability to respond to one another—that is, our *response-ability*.

It is this notion of ‘response-ability’ that sits at the core of commoning. Like Ingold [7], we borrow the phrase from the work of composer and musical theorist, John Cage. In a lecture titled *Experimental Music*, Cage [23] proposed that in order to truly listen to music, one needed to give up “the desire to control sound” and “set about discovering means to let sounds be themselves rather than vehicles for man-made theories or expressions of human sentiments” (p. 10). In other words, contrary to modern aesthetic sensibilities rooted in a hard empiricism ([3], ch. 9), listening to music, for Cage, was not a process of encoding or deciphering projected sounds, as if true meaning lay somewhere behind or beneath the specific notes being heard. But rather sound is unimpeded—it is *there*— “occupied with the performance of its characteristics” ([23], p. 14). To listen, then, is primarily a phenomenal, not physical, experience: meaning, it compels one to stretch towards the sound as it is, attending and responding to its characteristics; *feeling* its tone, frequency and amplitude [23]. Feeling, in this sense, is not haptic—physically responding to a thundering bass or screeching pitch—but experiential; an opening where one exposes themselves to the ‘goings on’ of sound from within. It is through this opening up and exposure where people become “response-able” —allowing the music, the sound—to move them, as they move it ([23], p. 10). Understood as such, listening would not be a one-way passage, receiving a sensory stimulus through the ear, to then be interpreted by some analytic device in the mind (i.e. an input and output). It is rather a *relation* with sound, in which one is moved not by a desire to control, manipulate, exploit or interpret, but by a genuine, active care for what they are directly attending and responding to, what they are *becoming-with*.

There is alignment here with the Gibsonian idea of direct perception, in which one actively engages in the physical act of perception in order to continuously interact with the environment [2]. This is also aligned with what we—the authors—understand by *experience*. It is not to be construed as a subjective attribution of meaning to stimuli from the world. Instead, experience is based on direct perception of the world as our most basic source of understanding reality and learning from our development. As ecological psychologist Edward Reed [24] eloquently emphasises, experience happens at the ecological level, in which we experience the world in terms of what it means for us, for our action and interaction, and is thus fundamentally collective.

It is in the attentive responsiveness of becoming *response-able* that our view of research as a sustainable practice in the sport sciences resides. Mentioned when discussing this paper’s inception, what one is required to become response-able to are the *experiences* of others. As Cage [23] suggested, being response-able to the experiences of others would not involve deducing a documented experience of another through a theoretical framework such that it can be interpreted. But it would be to *join with* their experience, carefully noting and responding to what they have to share, as they would to us. This process:

“[…] entails an attentive stretch whereby every participant casts their experience forward in ways they can answer to the experiences of others, and they likewise, so as to achieve a *correspondence* that goes *beyond* what any of them could have imagined at the outset, and that in turn allows them to *carry on* their lives together” ([7], p. 38)

Sustainability, then, is a *response-ability* we all have to the experiences of others—both human and nonhuman—that we go through life with. For in becoming response-able, we open ourselves to new beginnings, new opportunities for life to carry on, to developing experiences, thereby harbouring the conditions for a *sustained* growth of knowledge that transcends where we have been, but guides where we are going.

## Knowing is Ecological

Central to such a view of sustainability is a relational ontology—an appreciation that the world’s inhabitants are not discrete ‘objects’ destined to fulfil some prior established ‘potential’, but are entangled ‘things’ perpetually on the cusp of becoming [11, 25, 26]. It is this entanglement that sustains the growth of all living things, engendering the conditions for life to carry on [7]. In his seminal text *Art as Experience*, John Dewey [4] emphasised this wonderfully, stating that if one *really* wants to understand the flowering of plants, they must start with ‘the *interaction* of soil, air, water and sunlight that conditions the plants’ growth’ (p. 4, paraphrased). This is because, according to Dewey, for one to know the phenomena that has caught their attention, they need to ‘begin with it in the raw; in the events and scenes that arouse interest and enjoyment as they look and listen’ ([4], p. 3, paraphrased). Otherwise stated, they must immerse themselves in the natural ecology of what they seek to know, joining not as a passive occupant looking upon and documenting events, but as an active inhabitant looking within, primarily experiencing its coming-into-being.

What leads one to know through such inhabitation and continued correspondence is not recognition, but direct perception [4]. The contrast here is not vacuous and does require brief discussion. Notably, in recognition, perception is arrested, and we fall back “upon some previously formed scheme” that creates the basis of our observation ([4], p. 54). This means that there is no interaction between the observer and the observed, leading one to un-emotively and un-responsively know the world through the attachment of labels to various characteristics or qualities of what they are *looking at*. In the sport sciences, such a dominant model is apparent in research that seeks to ascribe the label of ‘talent’ to youth athletes based on putative physical or technical performance metrics, which are time-sensitive (confined to that moment of development), and reductionist (oft-measured in isolation, in test evaluation contexts far removed from performance conditions in which such characteristics occur) [27]. This snap-shot, one-sided approach leaves little room for the researcher to join with (i.e. get to know) the storied coming-into-being of individuals labelled as ‘talented’ or ‘non-talented’. They are viewed, instead, ‘objectively’ as if being categorically fixed and final in form [28]. Comparatively, in direct perception, there is what Dewey ([4], p. 54) refers to as an active ‘taking in’; “an act of reconstructive doing” where one becomes alive to the goings on of what interests them from within. This creates an inner commotion, a stirring that extends throughout one’s being that carries on; an *undergoing* that overflows to the next *doing*. Unlike recognition, there is an inevitable surrender here, a ‘giving up’ on the desire to control and label, replaced with a genuine curiosity that leads to the ongoing pick-up of things that may not have been seen, heard, tasted, or felt before. Such a dynamic opening up is indeed forward facing, as one is required to anticipate where next to move. This anticipation, though, is not a prediction of what is to come; it is a way of looking ahead so that one can remain open to new beginnings as they grow into a deeper, more attuned way of *knowing-in-becoming-with*.

James Gibson [2, 16], in his pioneering work on an ecological approach to visual perception, referred to this as knowledge *of* the environment. Such knowledge is direct and unmediated, grown through a progressive sensitivity to information omnipresent in an animal’s environment [2, 16]. It is the attunement of an animal’s entire perceptual system to the patterned structure of the invariant features of this information that directly specifies invitations to act available at that time [29]—that is, *affordances* ([2], ch. 9). Gibson [2] was rather emphatic in his claim that “to perceive an affordance is *not* to classify an object” (p. 134, emphasis added)—implying that to know *of* the world, it is not a prerequisite to ascribe labels to its constituents. Before we have the capacity to ascribe a label to an object, we have to perceive it, and according to Gibson, we perceive the object based on what we can do with it. Why this distinction in ecological psychology is important, is that it means that there is no limit to one’s knowledge *of* what interests them; “one can look as carefully as one wishes, and […] *always* discover *new* information” ([24], p. 94, emphasis added).

Contrastingly, second-hand information, or secondary experience, manifest in words, codes, pictures, data and symbols, represents knowledge *about* the environment [16]. Similar to Dewey’s account of recognition, such knowledge is mediated and indirect, bound to the categorical confines of what has been produced by another human individual. While indeed such second-hand information can be supportive in helping us know the world, it is one’s knowledge *of* their environment that directly regulates behaviour *in* it [2]. A nice example of this in science was shown in David Turnbull’s analysis of the TEA laser developed by Bob Harrison in the late 1960s. Specifically, Turnbull [30] noted that scientists outside of Harrison’s immediate group were unable to recreate the laser by using only second-hand information about its design found in published documents. To successfully recreate it, they had to *correspond* with the laser’s original makers, as what had not been considered in the published methodologies, were the nuanced differences between settings (i.e. laboratories) in which the laser was being built [30]. After all, “[s]cience”, declared Joseph Rouse ([31], p. 72), “is first and foremost knowing one’s way about in the laboratory (or clinic, field site)”. Stated differently, while both types of knowledge supported scientists in (re)creating the laser, it was knowledge *of* their surrounds that enabled them to harness knowledge *about* its design. Applied to contexts in sport, this would be reflective of verbal instructions provided by a coach—perhaps scribed onto a whiteboard—providing performers with information to know *about* an opposition prior to heading out to the performance area. But during performance, it would be the performers’ knowledge *of* their surrounds (i.e. seeing subtle changes in movements of opponents, hearing a teammate’s voice, or feeling changes in wind strength and direction) that directly regulates their behaviour as competition unfolds. This is why, to us, *knowing is ecological, not recognitive*; predicated on direct perception of our world, not systems of classification.

There are four points we wish to make regarding this proposition, each implicating our view of research practised sustainably in the sport sciences.^2^ First, knowledge grows through immersion, requiring one to *expose* themselves to the goings on of what interests them. Educational philosopher Jan Masschelein [32] notes that in such moments of exposure—from the Latin *ex-positio*—we are pulled out of our defensive positions, opening ourselves up to vulnerability and uncertainty. Indeed, this can be uncomfortable and unsettling, especially for those swimming in a scientific mainstream that idealises certainty and control. But absolute certainty is a mere falsity in a world suspended in motion; for every time we step forth, we put ourselves at risk to its emergent goings on. We are, as Tim Ingold ([33], p. 9) says, continually “falling-forwards”. What uncertainty offers, though, is an opportunity to become sensitive to what the world has to share with us, if would we just pay close attention. Think, for example, of when one loses their way while on a hike in the woods—it is here, in this moment of uncertainty where one becomes deeply attuned to the sounds, sights and smells of their surrounds, stretching towards subtle clues that could guide them along their way. For where one claims for certainty—strictly following a map or route prescribed for them—they turn themselves away from the infinite possibilities to get to know the world a little better. Knowledge, secondly, is *attentional*—it draws us out into the world so that we can respond to what it has to share with us [3]. This is not about searching inwardly for some putative mechanism of control, but about mixing with the coming-into-being of what draws our curiosity. Third, knowledge is not possessive—it is not an entity to be ‘acquired’, ‘gained’ or ‘transmitted’ [7, 34]. Rather, it grows in us and we grow into it by *dwelling*-in-place [35]. The knowledgeable sport scientist is one who is deeply embedded in the context of their study, aligning their perception to its ebbs and flows by carefully observing and participating with its becoming. Perhaps, then, knowing is not what you have, *but who you are as a fellow traveller in a world perpetually on the move*. Fourth, knowledge is *inexhaustive*, carrying on for as carefully as one attends to what interests them [3]. Comparatively, when one ascribes a label to an object, as in recognition, it risks being classified away, as if in its characterisation it is ‘done’, ‘complete’, ‘final’—it has nothing more to share, there is nowhere further to go. This is exactly why recognition is an *un*sustainable means of inquiry—it leads to dead-ends, a view of the world as a puzzle filled with pieces destined to be put in their ‘correct’ place. Understood inexhaustibly, though, the world would not be a puzzle to be solved, but would be a whirl, a vortex, a wave suspended on the cusp of crashing that presents infinite opportunities for continued wonder and astonishment. In other words, there would be no potential to fulfil, just the possibility to carry on. This is to turn answers into questions, further opportunities to join with, to know better. For research practised sustainably, knowledge is not a place to *destinate* (i.e. to reach a final point in a journey) or a turf to defend from others. It is, rather, how we become (and remain) continuously alive to what the world—entangled, dynamic, messy, abuzz—has to share with us, and we with it.

## Towards a Response-Able Scholarship in the Sport Sciences

**Fig. 1:**
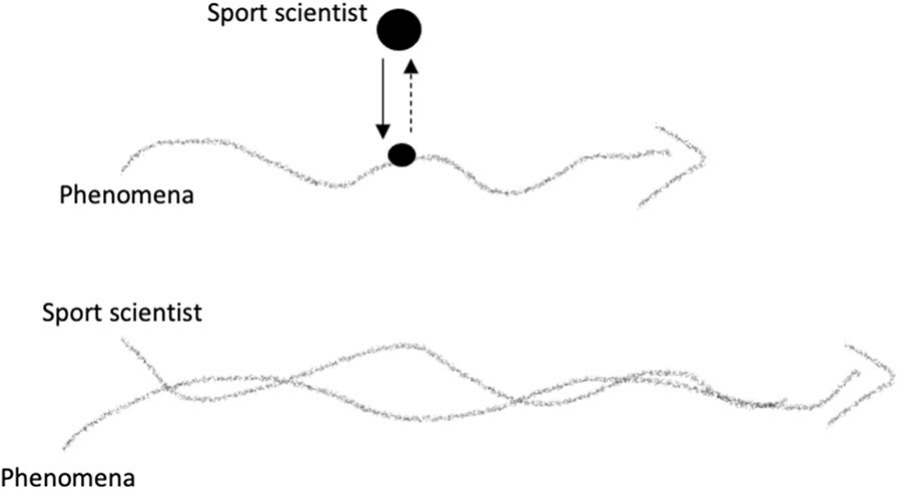
Research ‘done’ in a *vertically* integrated approach (top), contrasted to research ‘undergone’ *longitudinally* through attentive and responsive participant observation (bottom). For further insight into these contrasting approaches to research in the sport sciences, see Woods and Davids [35].

Indeed, the growth of such knowledge requires a *different* approach to inquiry, one that departs from the traditional hypothetico-deductive theory of the scientific method pervasive to the sport sciences [13, 14]. In this traditional method, knowledge, viewed (re)cognitively, is produced and integrated vertically, where “concepts and hypotheses, determined separate to the phenomena one is to study, sit above the goings on of the phenomena ‘at ground level’” ([11], p. 5–6). This leads to an aerial perspective of phenomena, where resulting observations are fed back up into a conceptual framework to be modelled, classified and labelled accordingly (Fig. 1). Sport scientists taking up with such an approach do not inhabit a place in-among the coming-into-being of phenomena, but occupy a space out-above its goings on [9–11]. It reflects our earlier Deweyan accounts of recognition, where the goal of one’s inquiry is to ‘properly’ label what is being looked at, viewed fixed in form and separate from the processes that gave rise to it [17]. This is exemplified in the sport sciences through research that ascribes the control and coordination of athlete movements to some putative internal model, scheme or representation (cf. [36]). Such a reduced and fragmented view immediately severs movement from the conditions and contexts that directly and continually shape and constrain it, thereby perpetuating *organismic asymmetries* in research and practice [15]. Why this is unsustainable, is that it blinkers sport scientists to the very processes that enable life to carry on. For example, if the goal of inquiry is to classify, label and categorise, then what room is left for growth, exploration, adaptation, improvisation and transformation? Moreover, if observations can simply be attributed to some *indirect* model or representation purportedly residing beneath what is being observed, then how responsive can the sport scientist be to what relevant phenomena have to share *directly* with them? There would be no conjoining of varied experience through commoning, as there would be no correspondence between the researcher and research. Instead, there would be a deliberate quietening of the phenomena, a distancing that ensures the researcher can profess their ‘objectivity’ about what they are *looking at* [9].

In searching for a response-able approach to inquiry, we found inspiration in the two-century old work of polymath, Johann Wolfgang von Goethe (1749–1832). Although known for poetry and other literary masterpieces, it is Goethe’s lesser-known approach to scientific inquiry that is of particular interest, given its grounding in a participatory, relational worldview [19]. To exemplify, in a Goethean approach to inquiry, knowledge growth is unbound, suspended in one’s *active participation with* what holds their attention [17]. As written in Goethe’s Faust, “in the beginning was the act”, which contrasts with the biblical St John’s Gospel’s, “in the beginning was the Word”. This contrast is meaningfully profound, as it emphasises that there is no fundamental point of creation hidden behind phenomena waiting to be ‘dug up’, but that phenomena are in perpetual creation, entangled within an ecology of relations of which ‘we’ are apart. It imbues the realisation that the phenomena we study are *always* richer than the mere abstractions, words and models typically used to explain them in the scientific mainstream [17, 19]. This is because phenomena encountered—like the flowering of a plant, the feeding of a bird, or working with an athlete finding their way through a practice task—have a ‘fullness’ in themselves; meaning, there is no need for their representation, they are ‘(t)here’ to be directly perceived in-becoming [17]. As shown in Fig. 1, research undergone in such a delicate way would not seek to represent or label objects looked down upon, but would encourage a perceptual attunement to its ebbs and flows, *joining with to follow along*. The former is vertical, the latter longitudinal, and why this directional contrast is important, is that it signals a shift in which participant observation goes beyond objectivity, truth beyond facts. For in allowing the phenomena into our being, we allow it to guide our attention,^3^ and it is in this moment of correspondence where ‘we’ become the very instrument for knowing^4^ [8, 9, 33].

## Entering into a Good Conversation^5^

In an exceptional paper titled *Doing Goethean Science*, Craig Holdrege [17] suggested that Goethe’s approach to inquiry could be viewed as *entering into a conversation with nature*. Think, for example, of when we enter into a conversation with someone or something that we are fond of, perhaps even one we love. In these conversations, we typically inquire about each other’s well-being, as we simply want to get to know each other better. As Goethe (cited in [39], p. 307, emphasis added) states somewhat radically, in such moments of correspondence, we want to “become utterly identical *with*”. What drives this inquiry, is *curiosity*—an interest that has been sparked about someone or something that we are drawn to attend to. As we inquire about this interest, we ask questions and they are asked of us—a ‘going along with’ emerges in which we attend and respond. Holdrege [17] suggests that such questions could be understood as ‘probes’ or ‘experiments’, not though, as the mainstream scientific approach would propose—i.e. used to prove or disprove a specific hypothesis determined *a priori*. Undertaken as such, a conversation would likely digress into a one-sided interrogation, where one speaks and the other is spoken to. This, we suggest, is synonymous with our earlier discussion of a vertically integrated (or perhaps vertically *interrogated*) approach to inquiry, where concepts are imposed down onto phenomena to be known about (see Fig. 1). In a co-responsive conversation, though, questions emerge from curiosity, asked with sincerity and responded to eloquently, because we *care* deeply for another’s well-being. There is an inevitable uncertainty when entering into such a conversation—we do not know where the questions asked will lead. The conversation may traverse old ground, but grow new insights and lead to deeper, richer perceptions of one’s surrounds. This uncertainty is what makes us attentive, actively *listening* to what the other has to share with us so that we can find ways to join with their experiences and carry the conversation on. Elsewhere, it has been suggested that this type of discourse is to take up with research as a journey, not a destination, where determinate pre-planning gives way to a progressively attuned responsiveness that sees the researcher selectively follow, and weave together, emergent lines of inquiry [40]. This is an approach to scientific inquiry that is undergone *together*, in which experiments are done *with*, not *on*, and observations are not *about*, but *with*.

Recall our opening quotation from Stephen L. Talbott—a good conversation is neither rigidly programmed or utterly chaotic. It resides somewhere in-between, in a metastable region that opens up the possibility to carry on in directions beyond what could have been broadly prescribed prior to. Indeed, this requires humility, as answers will likely lead to further questions, further opportunities to conjoin experiences as participants aspire to find in common [7]. This is risky, as entering into such a conversation imbues an obligation for one to traverse “webs that cannot be known in advance of venturing among their myriad threads” ([38], p. 132). But this is precisely what makes a Goethean approach to inquiry *sustainable*—there is no end to a good conversation, it is suspended in uncertainty, carried along and co-created by our response-ability.

This perspective is to appreciate that we—as sport scientists—are woven into the very fabric of the world’s coming-into-being; an appreciation that frees ‘us’ to *be with* the very phenomena which we want to know better. Stated differently, a good conversation cannot carry on—*it is unsustainable*—if participants do not jointly contribute to its becoming. Such a conversation is not static and documentational, it does not leave us unchanged after some immediate correspondence has passed. It is dynamic, improvisational, and transformational, as through our conjoining of experiences we grow, leading us to know the world, and perhaps the world us, a little better than before. Following Dewey [4], this is to appreciate that the *undergoing* of a conversation always overflows to the next *doing*, “to the extent that whatever you do next takes into itself something of the experience of what you did before” ([33], p. 7). A good conversation, then, has no ending, and by default, no beginning; it just carries on as we (re)search for ways of leading our lives, together. Residing here, is *hope*; a hope that those we *care* for, and are *curious* about, remain open—*response-able*—to the experiences we and they have shared, so that we can continue to get to know each other better. Thus, for research to be a sustainable practice in the sport sciences, we propose a *response-able scholarship ground in ongoing conversation*. As emphasised throughout this section, what carries this scholarship on—i.e. what makes it sustainable—is that it *cares*, it is *curious*, it is *open*, and it is *hopeful*.

## Conclusion

In this paper, we explored what it could mean for research in the sport sciences to be practised sustainably. To do so, we first framed sustainability through the notion of *commoning*—a conjoining of varied experiences to find ways of carrying on, together. This was to foreground the importance of our *response-ability* as sport scientists in coming to know the phenomena we study. In light of this perspective, knowing was suggested to be a fundamentally ecological process—meaning, it is the attuned perceptual system of the scientist responsive to the ebbs and flows of what captures their attention, that leads them to know better, not more. To guide the response-able practice of research in the sport sciences, we then wove in the work of Goethe. Given its relational and participatory worldview, Goethe’s ‘delicate empiricism’ encouraged an approach to scientific inquiry synonymous with that of entering into a good conversation. This philosophical perspective offered a different means of approaching inquiry in the sport sciences, a sustainable means that departed from the harder, traditional discourse of the empirical mainstream.

As mentioned in our paper’s introduction, what we have set out here is far from complete—there are many places yet to be explored, many good conversations still to be had. Perhaps, then, this paper has not answered a question *per se*, but exposed some loose ends, some threads that we have pulled, which if joined with, could lead to further questions. Indeed, in a dominant scientific discourse where certainty is the path and an ‘objective truth’ its destination, this may be uncomfortable for some. But this should not be a point of concern, as an ongoing line of questioning is precisely what makes our paper a manifestation of its very message. For as response-able sport scientists, we joined with the conversation opened by Hannah Arendt in her exceptional essay, *The Crisis in Education*. Moving this conversation along in our direction of travel meant that it was a *care* for our discipline, and where it could be heading in its journey, that led to our *curiosity* about what research practiced sustainably could mean in the sport sciences. Where we find ourselves now, is a place suspended in *hope* and *openness*—a hope that you reading these words are response-able, open to join your experiences with ours to carry the conversation on. Where this could lead us is unknown, but this uncertainty is the very point of a response-able scholarship. When the desire to control, predict, label and destinate—*to speak at and to*—gives way to genuine care, curiosity and hope, we can truly start to open ourselves from within. For in doing so, we may just find ways of carrying on, together.

## Data Availability

Not applicable

## Footnotes

1 For example, Exercise and Sport Science Australia (ESSA—Australia’s governing and accrediting body for exercise and sport scientists) have ‘sustainability’ as a ‘key principle’ underlining their recent strategic development plan. What this actually means, though—especially for research in sport science—is not well defined.

2 We draw inspiration for this section from the fourth chapter of Tim Ingold’s most recent book of essays, *Imagining for Real* [3].

3 Primatologist Shirley Strum ([37], p. 30) offers an exceptional account of such embodied communication when seeking to know baboons from a ‘baboon’s perspective’, noting: “I made a determined effort to forget everything I knew *about* how baboons are *supposed* to behave. Instead, I tried to let the baboons themselves “tell” me what was important” (emphasis added).

4 In her gripping book, *Staying With The Trouble*, Donna Haraway [38] presents an example of such a transformational shift in knowing-in-becoming-with. In a project called *PigeonWatch* that focused on learning how to live with-in multispecies entanglements, school children were enlisted to record the behaviours of local pigeons. In doing so, the children went from *labelling* them in patronising ways as “rats with wings” to *seeing* them as “social birds with lives and deaths.” In other words, through *attentively observing the pigeons in their becoming*, the children evolved from “bird hecklers and sometimes physical abusers to astute observers and advocates of beings whom they had not known how to see or respect. The schoolchildren became *response-able*” (pp. 24–25, emphasis added). What was central to the children’s emergent response-ability to the pigeons, was a care and curiosity that compelled them to join with, not look at. That is, they started knowing them *better* by directly perceiving them in *becoming-with*.

5 While alluded to in the forthcoming section, we wish to emphasise that the use of the word ‘conversation’ is not to be de-limited to a verbal interaction. From our ontological position, we can enter into a conversation with all things—animate and inanimate—granted we are attentive and (co)responsive to the ebbs and flows of our unfolding ‘conversation’. For example, I can enter into a conversation with a wave while surfing by attending and responding to its perpetual motions, as it responds to my motions while riding it. Like catching a good wave, the flow of this conversation is not vertical, but longitudinal, as the wave and I* become-with, while surfing along*.
